# In-Depth Phytochemical Profile by LC-MS/MS, Mineral Content by ICP-MS, and In-Vitro Antioxidant, Antidiabetic, Antiepilepsy, Anticholinergic, and Antiglaucoma Properties of Bitlis Propolis

**DOI:** 10.3390/life14111389

**Published:** 2024-10-29

**Authors:** Ebubekir İzol, Münire Turhan

**Affiliations:** 1 Bee and Natural Products R&D and P&D Application and Research Center, Bingöl University, 12000 Bingöl, Türkiye; 2 Faculty of Agriculture, Vocational School of Food, Agriculture and Livestock, Bingöl University, 12000 Bingöl, Türkiye; mturhan@bingol.edu.tr

**Keywords:** propolis, phytochemical content, antioxidant, antiglaucoma, anticholinergic, antidiabetic

## Abstract

Propolis is very significant in terms of its phytochemical content and biological activity among bee products. In this study, the antioxidant activities (total phenolic and flavonoid, Fe^3+^, Cu^2+^ (CUPRAC), Fe^3+^-TPTZ (FRAP) reducing, and DPPH^•^, ABTS^•+^ scavenging assays) of propolis collected from the Bitlis province of Türkiye were determined. In addition, the carbonic anhydrase I and II isoenzymes (hCA I and hCA II), α-glycosidase, acetylcholinesterase (AChE), and butyrylcholinesterase (BChE) inhibition activity and phytochemical profile of propolis and mineral content were determined by LC-MS/MS and ICP-MS, respectively. In propolis, 31 phytochemicals were found, and the highest concentration of acacetin (23.604 mg/g) was detected. It is seen that the phytochemicals in propolis provide antioxidant properties. The mineral content was screened for 18 elements and determined to be rich in the elements that make up the salt content. Total phenolic content was 215.14 mg GAE/g, and total flavonoid content was 79.11 mg QE/g. The Fe^3+^ reduction result was 0.940 (µg/mL), CUPRAC 1.183 (µg/mL), FRAP 0.963 (µg/mL), DPPH^•^ scavenging IC_50_: 16.7 (µg/mL), and ABTS IC_50_: 8.01 (µg/mL). hCA I enzyme inhibition results in IC_50_: 7.19 (µg/mL), hCA II 8.15, AChE 5.17, BChE 7.50, and α-Glycosidase 5.72. As a result of this study, it was determined that Bitlis propolis has high antioxidant properties and a rich phytochemical content. It was also observed that it is an effective enzyme inhibitor against epilepsy, glaucoma, Alzheimer’s, and diabetes, which are important diseases, and it can be evaluated in the treatment of these diseases and drug production.

## 1. Introduction

Propolis is a product formed by honeybees by collecting plant resins and mixing them with their own enzymes and beeswax. It is a sticky, resinous, fragrant substance in different colors, from dark yellow to brown, such as glue, used for many purposes in the hive [1,2,3,4]. Honeybees produce propolis for various purposes in the hive [5]. Propolis is used to close and repair the holes and cracks in the hive, to narrow the entrance of the hive or to isolate the hive from the outside environment, to mummify the harmful organisms that enter the hive, and to protect the colony from various bee diseases [6].

Propolis is a very significant bee product, with more than 300 components in its chemical content and high biological activity due to these components. This number is constantly increasing with scientific studies. Propolis content varies regionally but generally contains 5% pollen, 10% oil, 30% wax, 50% resin, and 5% simple carbohydrates, vitamins, and minerals [7]. Propolis has biological activities such as antioxidant, antiviral, antibacterial, antifungal, antiulcer, anti-inflammatory, antitumor, and immunostimulatory. Due to its biological activities, it can be used for various purposes in human and veterinary health, apitherapy, cosmetics, and pharmaceutical industries as a popular drug source. Due to these important properties, propolis has been added to the content of products in various forms, such as yogurt, fruit juice, cream, toothpaste, lotion, and tea. It has also become an important bee product with wide usage areas around the world [8,9].

Minerals are very significant with their different functions as catalysts in many reactions in biological systems [10,11]. Mineral deficiencies lie at the basis of most diseases. In addition, the excess of those with toxic properties causes poisoning and serious illnesses [12]. Therefore, it is necessary to determine the mineral content of consumed foods both qualitatively and quantitatively [13]. For this, inductively coupled plasma mass spectrometry (ICP-MS) technology provides the most sensitive and reliable results [14]. The chemical profile of propolis varies depending on ecological characteristics such as vegetation and the climate of the area where it was collected. Although this situation contributes to the diversity of propolis, it prevents propolis from having a certain chemical standardization and poses a problem in the quality control of propolis [15,16]. The reasons for the differences in propolis content are the preferences made by the bees and the plant resources in the region where the colony is located [17].

In studies on the antioxidant properties of propolis, it has been stated that there are phenolic compounds showing antioxidant properties in the content of propolis against free radicals, which are an important factor in biological activities [18]. Although the main component of propolis varies regionally, flavonoids have been reported to be over 25% in general [1,2,3,4]. It has been reported that these compounds show antioxidant activity and inhibit lipid peroxidation due to their free radical scavenging properties, and alternatively, they can be antioxidants due to their metal chelation formation [19]. In addition, it has been reported that propolis can be effective against oxidation in the food industry due to its high antioxidant content and can be used as an additive in human nutrition [20].

In recent scientific studies, it has been determined that antioxidants and enzymes are effective in the treatment of many different diseases. Antioxidants are pioneers in the treatment of many diseases by removing free radicals and preventing oxidative stress. They also do not cause diseases, as they do not form radicals themselves. Enzyme inhibitors are also used to cure many diseases. The carbonic anhydrase II (hCA II) enzyme was found to be associated with glaucoma, carbonic anhydrase I (hCA I) with epilepsy, acetylcholinesterase (AChE), and butyrylcholinesterase (BChE) with Alzheimer’s disease, and the α-glycosidase enzyme with diabetes [21,22,23,24,25]. That’s why it is very significant to determine the phytochemical content of propolis, a natural bee product, the inhibition potential of these enzymes, and their antioxidant properties [26,27,28].

Among the reasons for the study of Bitlis propolis are (1) Bitlis province is a place with important beekeeping activities; (2) Bitlis province has a rich flora; and (3) the parameters in this study have not been studied in the literature.

This study is the first to investigate the phytochemical profile by LC-MS/MS, mineral content by ICP-MS, some inhibition of metabolic enzymes, and antioxidant properties of propolis derived from Bitlis, a significant beekeeping city in Türkiye.

## 2. Material and Method

### 2.1. Chemicals

Standard phytochemical substances were purchased from Sigma-Aldrich (Steinheim, Germany) for use in LC-MS/MS and ICP-MS analysis. Commercial purchases of standards and other chemicals were made from Sigma-Aldrich Chemie (Steinheim, Germany) for use in antioxidant and enzyme assays. Using human erythrocytes and the Sepharose-4B tyrosine-sulfanilamide affinity column method, the carbonic anhydrase I (hCA I) and carbonic anhydrase II (hCA II) enzymes were highly purified.

### 2.2. Preparation of Propolis

Propolis was collected from Bitlis province, where Türkiye has important beekeeping activities. A propolis sample was obtained from *Apis mellifera* species. *Apis mellifera caucasica* and *Apis mellifera anatoliaca* species are generally found in this region. After freezing at −80 °C, this is because propolis is not affected by temperature, and the grinding process is better; it was ground with a grinder. Then, 30 mL of 70% ethanol solvent was added to 10 g of propolis and mixed for 3 days, and extraction was completed by the maceration method. The maceration method was chosen because it is one of the most preferred methods. A stock solution at a concentration of 10 mg/mL was prepared from the extracts that were filtered and dried thoroughly. Analyzes were made with this prepared stock solution.

### 2.3. Determination of Comprehensive Phytochemical Profile by LC-MS/MS

The phytochemical content of stock solutions prepared from propolis extracts was determined by LC-MS/MS using the method validated in previous studies [29]. In this assay, a Shimadzu-Nexera model ultra-high performance liquid chromatography (UHPLC) coupled with a tandem mass spectrometer was used for the quantitative analysis of 53 phytochemicals. LC-MS/MS instrument specifications, analysis conditions, and other data related to the analysis are as follows: LC-MS/MS, Shimadzu LCMS-8040 model; software, LabSolutions (Shimadzu, Kyoto, Japan); columns, RP-C18 Inertsil ODS-4 (100 mm × 2.1 mm, 2 µm) Agilent (Santa Clara, CA, USA) Poroshell 120 EC-C18 model (150 mm × 2.1 mm, 2.7 µm); mobile phase, acetonitrile and methanol; solvent flow rate, 0.5 mL/min; injection volume, 5 µL; MS operating conditions, nebulizing gas (N_2_) flow, 3 L/min; drying gas (N_2_) flow, 15 L/min; DL temperature, 250 °C; interface temperature, 350 °C, and heat block temperature, 400 °C. Full details of the method are available in the relevant reference [29].

### 2.4. Determination of Mineral Content by ICP-MS

Mineral composition was determined by modifying the method of Izol et al. [11]. First, a CEM-brand MARS6 ONE TOUCH (Bingöl, Türkiye) microwave extractor was used to solubilize the dried propolis using ultra-pure nitric acid. Samples that were fully dissolved were diluted using a 1% super-pure nitric acid solution in ultrapure water. Different concentrations of calibration solutions for ICP-MS were developed. For elemental analysis, ICP-MS NexION 2000 C (PerkinElmer Inc., Waltham, MA, USA) was utilized. Furthermore, ICP-MS calibration was completed before every experiment. The elemental analysis and calibration graph were checked using internal standards ^45^Sc and ^115^In. The elements Na, Mg, Al, K, Ca, V, Cr, Mn, Fe, Co, Ni, Cu, Zn, As, Ag, Cd, and Pb were examined by ICP-MS using Syngistix software version 2.2 following sample preparation and calibration graph creation (Kandemir et al. 2021 [12]). Analytical parameters related to the method are given in Table 1.

### 2.5. Total Phenolic and Flavonoid Contents

According to a prior study [30], the total phenolic and flavonoid contents of the propolis extract were determined. The propolis extract (0.5 mL) was combined with the Folin-Ciocalteu solution (1.0 mL) and Na_2_CO_3_ (0.5 mL, 1%) to determine the total phenolic content. After 2 h at room temperature, the mixes’ absorbance was measured at 725 nm. Total phenolic content was measured as milligrams of gallic acid equivalent (GAE), which was used as a standard [31].

The extract (0.5 mL) was combined with aluminum chloride (1.5 mL, 10%), ethanol (1.5 mL, 95%), distilled water (2.3 mL), and potassium acetate (0.5 mL, 1.0 M) to determine the total flavonoid concentration. After incubating the mixtures for 30 min at room temperature, the absorbance of the mixtures was measured at 415 nm. Total flavonoid levels were expressed as milligrams of quercetin equivalent (QE) per gram of propolis extract, and quercetin was utilized as a standard [32].

### 2.6. Radical Scavenging Assays

#### 2.6.1. ABTS^•+^ Scavenging Ability Assays

The ABTS^•+^ scavenging ability of propolis extracts was realized according to the Re et al. method [33]. Briefly, an aqueous solution of ABTS (7.0 mM) was oxidized by K_2_S_2_0_8_ (2.5 mM) to produce the radical cation (ABTS^•+^). A phosphate buffer (0.1 M, pH 7.4) was used to dilute the ABTS^•+^ solution before use, and the absorbance value of the control was calibrated to 0.750 ± 0.025 at 734 nm. Then, 3 mL of propolis stock solutions were mixed with 1 mL of ABTS^•+^ solution at various concentrations (20–60 μg/mL). At 734 nm, the absorbance of ABTS^•+^ was measured following a 30-min incubation period [34,35].

#### 2.6.2. DPPH^•^ Scavenging Ability Assays

Propolis’s DPPH^•^ scavenging activities were assessed using the Blois assay [36]. For this purpose, 1 mL of DPPH^•^ solution (0.1 mM) with blue color prepared in ethanol was added to propolis stock solutions at different concentrations (20–60 μg/mL). It was then incubated at room temperature for 30 min, and absorbance values were recorded at 517 nm [37].

The radical capture potential (RSC) of propolis extract was calculated using the formula: RSC (%) = (1 − Ac/As) × 100, where Ac is the absorbance of the control and as is the absorbance value of the sample. In addition, the IC_50_ values of the same samples were obtained from the graphs as μg/mL [38].

### 2.7. Reducing Ability Assays

#### 2.7.1. Cu^2+^ Reducing Ability (CUPRAC)

The Cu^2+^ reducing capacity of propolis extracts was determined by a modification of the Apak method [39]. Firstly, propolis stock solution was prepared at different concentrations (10–30 µL), and standard antioxidant solutions were added to the tubes. Then, respectively, 125 µL of CuCl_2_, 125 µL of neocuprine solutions, and 125 µL of CH_3_COONa buffer solution were transferred. Distilled water was poured into the tubes until a total volume of 1000 µL was reached. Lastly, absorbance values at 450 nm were measured following a half-hour of darkness [40].

#### 2.7.2. Fe^3+^-Fe^2+^ Reducing Ability

The calculation of the Fe^3+^-Fe^2+^ reducing capacity of propolis extracts was made with the modification of the Oyaizu assay [41,42]. Propolis extracts were used to create standard antioxidant solutions at various doses (40–120 µL) and a 1 mg/mL stock solution for this purpose. One milliliter of phosphate buffer and 750 µL of distilled water were added to each prepared test tube. Each tube was then filled with 1 mL of [K_3_Fe(CN)_6_] (1%) and left in the dark (40 °C) for 30 min. Following the incubation period, 250 mL of FeCl_3_ (0.1%) and 1 mL of TCA (10%) were added to the tubes and swirled. Lastly, measurements of absorbance values at 700 nm were made [43,44].

#### 2.7.3. Fe^3+^-TPTZ Reducing Ability (FRAP)

Propolis stock and standard solutions prepared at different concentrations (20–60 μL) in the Fe^3+^-TPTZ complex reducing ability method were transferred to test tubes [45,46]. Then the volumes of these tubes were made up to 500 μL with buffer solution, and 2250 μL FeCl_3_ and 2250 μL FRAP solutions were added to each of the tubes. Following a half-hour of dark incubation, tubes totaling five milliliters were mixed with vortex, and the absorbance values were measured at 593 nm [47].

### 2.8. Enzyme Inhibition Assay

#### 2.8.1. hCA I and hCA II Enzyme Inhibition Assay

In using sepharose-4B-tyrosine-sulfanilamide affinity chromatography, the hCA I and hCA II isoenzymes were extracted from human erythrocyte cells for this study. In our prior study [48,49], the methodology is described. At 25 °C, 3 min, and 348 nm, acetazolamide, a common CA inhibitor, and its stock solution were tested [50]. The CA inhibition potential of the extracts was calculated using an activity (%)-compound plot. Based on the activity (%) versus the compound plot, the IC_50_ value was determined.

#### 2.8.2. AChE and BChE Enzyme Inhibition Assay

The commercial supplier of the AChE and BChE utilized in this investigation was Sigma-Aldrich. Studies on the inhibition of these enzymes involved in the cholinergic breakdown processes and butyrylcholine were carried out using the Ellman et al. [51]. assay established. The substrates that were utilized were acetylthiocholine iodide (AChI), butyrylthiocholine iodide (BChI), and 5,5′-dithiobis(2-nitrobenzoic acid) (DTNB). Following the preparation of the reaction contents in the control and sample tubes at various concentrations, spectrophotometric measurements were taken at 412 nm. Based on the collected data, IC_50_ values and inhibition types were identified.

#### 2.8.3. α-Glycosidase Enzyme Inhibition Assay

These substances were tested using p-nitrophenyl-D-glycopyranoside (p-NPG) as a substrate to determine if they may inhibit the α-glycosidase enzyme’s activity [52]. Initially, 40 µL of the sample solution was mixed with 200 µL of phosphate buffer (0.15 EU/mL, pH 7.4). Moreover, 50 µL of p-NPG in phosphate buffer (pH 7.4, 5 mM) was added after preincubation, and the mixture was incubated once more at 30 °C. An earlier study used spectrophotometry to quantify absorbance at 405 nm.

### 2.9. Statistical Analysis

For every sample, every experiment is carried out three times. The results were analyzed using one-way ANOVA and Tukey’s post hoc test; *p* < 0.05 was considered statistically significant. The data are displayed as the mean ± SD (*n* = 3).

## 3. Results and Discussion

### 3.1. Phytochemical Profile Results by LC-MS/MS

The results of the quantitative phytochemical content by LC-MS/MS of the propolis sample are given in Table 2. Since method validation data and analytical parameters are given in the reference method [29], only molecular ions of the standard analytes (*m*/*z* ratio), retention time, coefficient of determination, fragment ions, limit of quantification and limit of detection, and concentrations of propolis are given in this Table 2.

**Figure 1:**
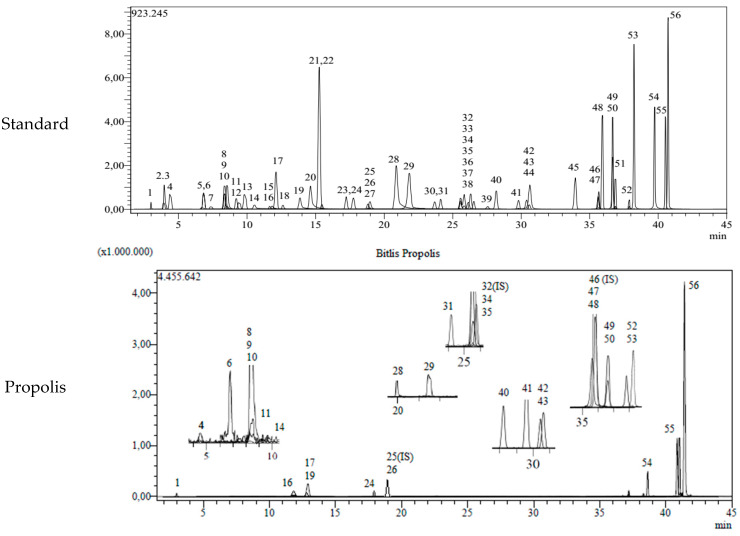
LC-MS/MS chromatogram of standard phytochemicals and propolis.

LC-MS/MS chromatograms of standard phytochemicals and propolis are given in Figure 1.

The phytochemical content of the propolis was found to be rich. Quantitatively, the component acacetin (23.604 mg analyte/g extract) was determined at the highest concentration in propolis. Significant bioactive phytochemicals found in propolis are as follows: naringenin 6.56 (mg/g), quinic acid 4.89 (mg/g), ferulic acid 4.378 (mg/g), chrysin 3.613 (mg/g), *p*-coumaric acid 2.735 (mg/g), caffeic acid 1.839 (mg/g), and kaempferol 1.286 (mg/g). Table 3 provides the chemical formula for these substances. In the literature, it has been reported that acacetin is effective against cardiovascular diseases, inflammation, infections, and some types of cancer and prevents lung damage and arthritis. It has also been shown to have antioxidant, anticancer, anti-inflammatory, antibacterial, antiviral, and anti-obesity properties [53]. Naringenin flavonoid has antioxidant, antiinflammatory, antiviral, antitumor, and antibacterial properties. It also strengthens intracellular signaling against insulin concentration by sensitizing hepatocytes to insulin, which is important for diabetic patients [54,55]. Quinic acid, a carboxylic acid, shows antioxidant, anticancer, antidiabetic, antiviral, and antimicrobial properties [56]. Ferulic acid is known as a powerful membrane antioxidant and is used in foods to prevent lipid peroxidation. The phenoxy and hydroxy groups in its structure remove free radicals and thus show therapeutic effects against diseases [57]. The antioxidant properties of propolis may be due to these significant phytochemicals it contains.

### 3.2. Mineral Content Results by ICP-MS

The elements Na, Mg, Al, K, Se, Ca, V, Cr, Mn, Fe, Co, Ni, Cu, Zn, As, Ag, Cd, and Pb were quantitatively determined by ICP-MS of Bitlis propolis. The results are given in Table 4. The highest concentrations of K (1521 mg/kg), Fe (1068 mg/kg), Al (1066 mg/kg), and Mg (710 mg/kg) were determined, while toxic elements such as As, Ag, Cd, and Se were not determined. In general, propolis was found to be rich in elements with salt components.

Minerals are significant in many reactions as catalysts in biological systems. Fe and Cu are also widely used in antioxidant property determination assays. In addition, deficiencies of some minerals cause important diseases, while high concentrations of others cause poisoning. Therefore, the importance of minerals is quite high.

The difference in the mineral components of propolis is due to the mineral content of the source plants as well as soil structure and climatic conditions. In a study conducted in Morocco, the mineral content of 20 different propolis samples was determined with 13 macro and micro elements. The highest concentration of Ca (1325 mg/kg) was determined, and Cd, Cr, Co, and Ni were not detected in all samples [58]. In this study, the highest concentration of K (1521 mg/kg) was determined, and As, Ag, and Se were not detected. Elements that were not detected in Moroccan propolis were determined at low concentrations in Bitlis propolis.

In another study, the mineral content (Ca, Cd, Fe, K, Mn, Zn, Cu, Hg, and Pb) of 31 propolis samples from Northern Spain was identified by ICP-MS. As a result, Na was found to be below the limit of determination (50 mg/kg) in all samples, with the highest concentration of K (1690 mg/kg) on average. Toxic Pb (average 4056 µg/kg), Hg (average 5.9 µg/kg), and Cd (26.5 mg/kg) were detected [59]. The highest K was observed in propolis from Spain and propolis from Bitlis. Pb was found to be 4056 µg/kg on average in Spanish propolis and 18.6 µg/kg in Bitlis propolis. Bitlis propolis was observed to contain much lower Pb than Spanish propolis. These studies show that different vegetation, soil, and climate affect the mineral content of propolis.

### 3.3. Total Phenolic and Flavonoid Content Accounting

**Figure 2:**
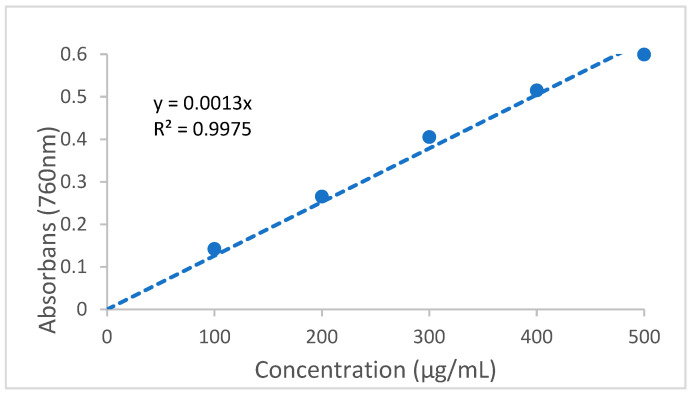
Total phenolic content standard calibration curve graph.

**Figure 3:**
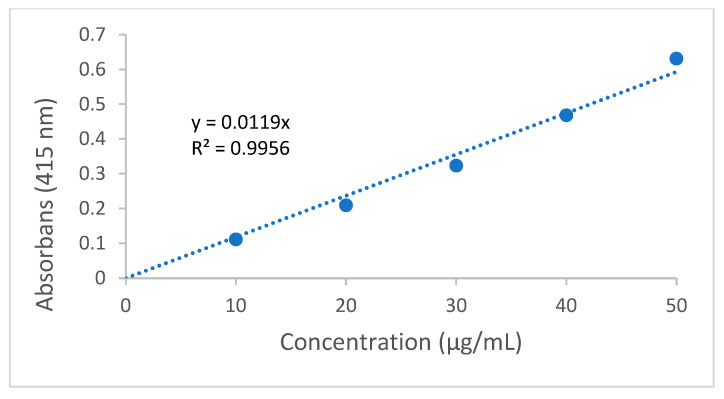
Total flavonoid content standard calibration curve graph.

The results of the total phenolic and total flavonoid content of propolis samples are given in Table 5. The total phenolic content standard calibration graph is given in Figure 2. The total flavonoid content standard calibration graph is given in Figure 3.

### 3.4. Antioxidant Results

The results of antioxidant-reducing assays of propolis are given in Table 6.

According to the Fe^3+^-Fe^2+^ reducing assay, the antioxidant capacity of propolis was found to be below the standard antioxidants. However, propolis showed higher antioxidant activity than trolox and tocopherol standards in the CUPRAC assay, and trolox, BHT, and BHA showed higher activity than standard antioxidants in the FRAP assay. The highest antioxidant property of propolis was observed in the CUPRAC assay (1.183 µg/mL). Thus, it was determined that propolis showed high antioxidant properties by in vitro spectrophotometric reducing assays.

Table 7 lists the outcomes of DPPH^•^ and ABTS^•+^ scavenging assays performed on the extract.

In DPPH and ABTS scavenging assays, it was determined that propolis showed lower activity than standard antioxidants. However, it was observed that propolis showed antioxidant properties close to the BHT standard in the DPPH test and close to the α-tocopherol standard in the ABTS assay.

### 3.5. Enzyme Inhibition Results

#### 3.5.1. hCA I and hCA II Enzyme Inhibition Results (Antiepilepsy and Antiglaucoma Properties)

Antiepilepsy property of propolis sample was determined by inhibition of hCA I isoenzyme, and antiglaucoma property was determined by inhibition of hCA II isoenzyme. Enzyme inhibition results are given in Table 8.

As a result of the inhibition of hCA I and hCA II isoenzymes in propolis, it was determined that it showed lower inhibition compared to the standard inhibitor. However, it was determined that propolis inhibited these two enzymes and showed antiepilepsy and antiglaucoma properties.

#### 3.5.2. AChE, BChE, and α-Glycosidase Enzyme Inhibition Results (Anticholinergic and Antidiabetic Properties)

The anticholinergic property of propolis was revealed by AChE and BChE enzyme inhibition, and its antidiabetic property was revealed by α-Glycosidase enzyme inhibition. Enzyme inhibition results are given in Table 9.

The α-glycosidase enzyme inhibition of propolis was found to be lower than the standard. However, AChE and BChE enzyme inhibitions were higher than the standard. Thus, it was determined that propolis has antidiabetic and anticholinergic properties, especially its anticholinergic property, which is high.

Phytochemicals are considered precursor components in many biological activities. It has a very important effect, especially on human health [47]. Since propolis is a substance formed by bees with plant extracts collected by bees and their enzymes, its phytochemical content is seen as the main reason for its biological activities [60]. Especially its antioxidant properties are interpreted with its phytochemical content. It is determined by studies that propolis has high antioxidant activity and phytochemical content in general [61]. Propolis shows positive effects on human health with its rich phytochemical content and increases the chemical stability of foods by preventing oxidation with its antioxidant activity [60,62].

In a study, the total phenolic content of ethanolic propolis extracts obtained from propolis samples obtained from different parts of Türkiye was determined to be 88.7–261.1 mg GAE/g, and the total flavonoid content was 37.5–150.4 mg QE/g [63]. In a different study, the total phenolic and total flavonoid content of aqueous propolis extract was determined to be 124.3 mg GAE/g and 8.15 mg QE/g [64]. In another study, the total phenolic contents of Ankara propolis (8.50 mg GAE/g) and Giresun propolis (7.88 mg GAE/g) were determined [65]. In different studies, the total phenolic content of propolis was determined as follows: 302 mg GAE/g [66], 299 mg GAE/g [67], 212.7 mg GAE/g [68]. In this study, total phenolic substance content was found to be 215.14 mg GAE/g, and total flavonoid content was found to be 79 mg QE/g. As a result of this study, Bitlis propolis was found to contain more phenolic substances than propolis obtained from different places. The amount of flavonoid substance was found to be different when compared with other propolis. The high total phenolic substance content of Bitlis propolis can be explained by vegetation, geography, climate, and bee species.

In the study in which antioxidant activities of Berdav propolis were determined, ABTS^•+^, DPPH^•^ scavenging activities and Fe^3+^ reducing, CUPRAC, and FRAP reducing capacities were found to be IC_50_: 8.15 (µg/mL), IC_50_: 20.55 (µg/mL), 1.545 (µg/mL), 2.323 (µg/mL), and 1.755 (µg/mL), respectively. In this study, the phytochemical content of Berdav propolis was determined by the same method. 26 of 53 different components were quantitatively determined. The component determined at the highest concentration was acacetin (76.359 mg analyte/g propolis) [48]. When the biological activities and phytochemical contents of Bitlis propolis and Berdav propolis were compared, it was observed that Bitlis propolis showed slightly higher antioxidant properties than Berdav propolis, and enzyme inhibition results were found to be close to each other. In Bitlis propolis, hCA I and BChE enzyme inhibitions were also studied. When the phytochemical contents of the two propolis were compared, it was observed that Bitlis propolis contained more components, but Berdav propolis contained a higher concentration of components. Thus, it is thought that different propolis can show different chemical content and biological activity, and each geography should be investigated as a different substance. In these studies, Anatolian propolis was found to have high antioxidant properties. For this reason, it is seen that Anatolian propolis can lead to natural products with high antioxidant properties that are pioneers in the treatment of diseases. With this study, it is predicted that Bitlis propolis will be therapeutic for patients with Alzheimer’s, epilepsy, diabetes, and glaucoma.

In the study investigating the biological activity of propolis collected from Erzurum province of Türkiye, the Fe^3+^ reducing result was 0.568 µg/mL, the CUPRAC result was 0.814 µg/mL, DPPH^•^ scavenging was 31.8 µg/mL, and ABTS^•+^ scavenging was 14.2 µg/mL. In that study, no enzyme inhibition was performed, and 13 different phenolic acid components were analyzed by LC-MS/MS [64]. It was observed that Bitlis propolis showed higher antioxidant activity than Erzurum propolis. In a study on four different Nigerian propolis, the average DPPH^•^ scavenging activity was determined to be 158.5 µg/mL [69]. It was determined that Bitlis propolis showed much higher DPPH^•^ scavenging activity (16.7 µg/mL) than Nigerian propolis. Similar studies have shown that the chemical composition of propolis is quite rich [70]. As a result of these studies, it was proven once again that propolis from different regions showed different antioxidant activity.

In the study in which the antioxidant properties and chemical content of Yalova propolis samples extracted with different solvents containing more than 300 different components were investigated by LC-MS/MS, the total phenolic (112.08) and total flavonoid (47.54) contents of 70% ethanolic extracts were determined. In addition, antioxidant potentials were determined by the CUPRAC, ABTS, and FRAP methods [52]. In general, Bitlis propolis was found to contain more total phenolic and flavonoid substances than Yalova propolis. In the study, 17 chemicals were screened, and the most prominent compound was determined to be chrysin. Chrysin was determined in Bitlis propolis at higher concentrations than other phytochemicals. However, the highest amount of acacetin was found.

In the study in which Anatolian propolis was investigated, total phenolic and flavonoid results of 40 propolis samples from different regions were found to be different. In addition, antioxidant properties were determined by FRAP and DPPH methods, and all regions were observed to be different. In addition, 25 phenolic components were screened, and 15 components were identified [71]. Gallic acid, protocatechuic acid, chlorogenic acid, syringic acid, 4-OH benzoic acid, rutin, epicatechin, and daidzein, which are phytochemical components screened in common with Bitlis propolis, were not detected in 40 propolis samples. However, gallic acid, protocatechuic acid, chlorogenic acid, and 4-OH benzoic acid were identified in Bitlis propolis. Therefore, it was seen that Bitlis propolis from 40 samples from seven different regions of Türkiye was different in terms of these components. Another reason for the detection of these components in Bitlis propolis may be the analysis method used. Because LC-MS/MS can analyze more precisely than HPLC. It was also determined that the antioxidant properties of Bitlis propolis and 40 different propolis were different, and Bitlis propolis showed higher antioxidant properties than most of them. In a study conducted in the Bitlis, 770 plant species were identified and the three largest families were *Poaceae* (77 taxa), *Fabaceae* (101 taxa), and *Asteraceae* (109 taxa). The largest genera were *Silene* L. (18), *Astragalus* L. (22) and *Trifolium* L. (27). The climatic conditions of the region are humid and very cold [72]. Since the properties of propolis vary according to flora and climatic conditions, flora, and climate information of Bitlis province was given.

In this study, the antioxidant activity of propolis samples from Bitlis province was determined by seven different methods, together with total phenolic and flavonoid amounts. The literature review determined that such a comprehensive study was almost nonexistent, and other studies used 2–3 methods. In addition, since there is no hCA I, hCA II, AChE, BChE, and α-glycosidase enzyme inhibition feature in the literature for Bitlis propolis, the antiepilepsy, antiglaucoma, antidiabetes, and anticholinergic potential of this propolis has been introduced to the literature for the first time. Determining the phytochemical content of propolis by LC-MS/MS and the fact that it is a validated method including 53 different components make this study important. In total, 31 bioactive phytochemicals were determined in the samples, and it was determined that propolis may be a raw material for an important bioactive component such as acacetin. It was observed that the antioxidant properties of propolis may be due to the high concentrations of acacetin, naringenin, quinic acid, ferulic acid, chrysin, *p*-coumaric acid, caffeic acid, and kaempferol phytochemicals. This study once again demonstrated that propolis samples from different geographies may have different biological activity and chemical content. In addition, according to similar studies, the reasons for the differences in antioxidant activities, enzyme inhibition properties, and chemical contents of propolis may be plant flora, climate structure, bee health, extraction method, and temperature conditions.

## 4. Conclusions

Propolis is a very valuable substance among bee products. As a result of scientific studies, it has high pharmacological and biological activity. As a conclusion, it was determined that Bitlis propolis has high antioxidant properties compared to propolis obtained from different geographies, especially Anatolia. It contains 31 different bioactive phytochemicals and can be a raw material for acacetin, naringenin, quinic acid, ferulic acid, chrysin, p-coumaric acid, caffeic acid, and kaempferol, which were detected at higher concentrations than other components. It was identified that the mineral content was rich in elements that are salt components and had high nutritional properties. Due to its strong enzyme inhibition properties, its potential against important diseases such as epilepsy, glaucoma, Alzheimer’s, and diabetes was also observed. Thus, it was shown that Bitlis propolis has healing potential against many diseases, but in vivo and clinical experiments are required to explain the metabolism.

## Figures and Tables

**Table 1:** ICP-MS analytical parameters.

Element	Linear Range	Correlation Coefficient (r^2^)	Limit of Detection (LOD)	Limit of Quantification (LOQ)
Na	0–25 (mg/kg)	0.9992	0.029	0.088
Mg	0–25(mg/kg)	0.9995	0.045	0.136
Al	0–25 (mg/kg)	0.9999	0.709	2.148
K	0–25 (mg/kg)	0.9992	0.022	0.067
Ca	0–25 (mg/kg)	0.9999	0.068	0.206
V	0–200 (μg/kg)	0.9999	0.283	0.860
Cr	0–200 (μg/kg)	0.9999	0.190	0.576
Mn	0–10 (mg/kg)	0.9999	0.249	0.754
Fe	0–10 (mg/kg)	0.9999	1.271	3.853
Co	0–200 (μg/kg)	0.9999	0.278	0.843
Ni	0–200 (μg/kg)	0.9999	0.334	1.013
Cu	0–200 (μg/kg)	0.9997	0.621	1.883
Zn	0–10 (mg/kg)	0.9999	0.301	0.914
As	0–200 (μg/kg)	0.9999	0.317	0.962
Se	0–200 (μg/kg)	0.9999	0.121	0.377
Ag	0–200 (μg/kg)	0.9999	0.128	0.387
Cd	0–200 (μg/kg)	0.9999	0.217	0.659
Pb	0–200 (μg/kg)	0.9999	0.725	2.197

LOD and LOQ concentrations of Na, Mg, K, and Ca are given in mg/kg; other elements are given in μg/kg.

**Table 2:** Results of phytochemical components by LC-MS/MS of propolis sample (mg analyte/g extract).

No	Analyte	Propolis	R.T.	M.I. (*m*/*z*)	F.I. (*m*/*z*)	r^2^	LOD/LOQ (µg/L)
1	Quinic acid	4.89 ± 0.18	3.0	190.8	93.0	0.996	25.7/33.3
2	Fumaric aid	-	3.9	115.2	40.9	0.995	135.7/167.9
3	Aconitic acid	-	4.0	172.8	129.0	0.971	16.4/31.4
4	Gallic acid	0.03 ± 0.0003	4.4	168.8	79.0	0.999	13.2/17.0
5	Epigallocatechin	-	6.7	304.8	219.0	0.998	237.5/265.9
6	Protocatechuic acid	0.152 ± 0.005	6.8	152.8	108.0	0.957	21.9/38.6
7	Catechin	-	7.4	288.8	203.1	0.999	55.0/78.0
8	Gentisic acid	0.085 ± 0.001	8.3	152.8	109.0	0.997	18.5/28.2
9	Chlorogenic acid	0.026 ± 0.0005	8.4	353.0	85.0	0.995	13.1/17.6
10	Protocatechuic aldehyde	0.096 ± 0.003	8.5	137.2	92.0	0.996	15.4/22.2
11	Tannic acid	0.058 ± 0.001	9.2	182.8	78.0	0.999	15.3/22.7
12	Epigallocatechin gallate	-	9.4	457.0	305.1	0.999	61.0/86.0
13	Cynarin	-	9.8	515.0	191.0	0.999	5.8/9.4
14	4-OH Benzoic acid	0.159 ± 0.003	10.5	137,2	65.0	0.999	68.4/88.1
15	Epicatechin	-	11.6	289.0	203.0	0.996	139.6/161.6
16	Vanilic acid	0.422 ± 0.006	11.8	166.8	108.0	0.999	141.9/164.9
17	Caffeic acid	1.839 ± 0.027	12.1	179.0	134.0	0.999	7.7/9.5
18	Syringic acid	-	12.6	196.8	166.9	0.998	82.3/104.5
19	Vanillin	0.126 ± 0.001	13.9	153.1	125.0	0.996	24.5/30.4
20	Syringic aldehyde	-	14.6	181.0	151.1	0.999	19.7/28.0
21	Daidzin	-	15.2	417.1	199.0	0.996	7.0/9.5
22	Epicatechin gallate	-	15.5	441.0	289.0	0.997	19.5/28.5
23	Piceid	-	17.2	391.0	135/106.9	0.999	13.8/17.8
24	*p*-Coumaric acid	2.735 ± 0.053	17.8	163.0	93.0	0.999	25.9/34.9
25	Ferulic acid-D3-IS	N.A.	18.8	196.2	152.1	N.A.	N.A.
26	Ferulic acid	4.378 ± 0.079	18.8	192.8	149.0	0.999	11.8/15.6
27	Sinapic acid	-	18.9	222.8	193.0	0.999	65.2/82.3
28	Coumarin	0.019 ± 0.0007	20.9	146.9	103.1	0.999	214.2/247.3
29	Salicylic acid	0.068 ± 0.001	21.8	137.2	65.0	0.999	6.0/8.3
30	Cyranoside	-	23.7	447.0	284.0	0.997	12.1/16.0
31	Miquelianin	0.015 ± 0.0003	24.1	477.0	150.9	0.999	10.6/14.7
32	Rutin-D3-IS	N.A.	25.5	612.2	304.1	N.A.	N.A.
33	Rutin	-	25.6	608.9	301.0	0.999	15.7/22.7
34	isoquercitrin	0.108 ± 0.002	25.6	463.0	271.0	0.998	8.7/13.5
35	Hesperidin	0.121 ± 0.004	25.8	611.2	449.0	0.999	19.0/26.0
36	*o*-Coumaric acid	-	26.1	162.8	93.0	0.999	31.8/40.4
37	Genistin	-	26.3	431.0	239.0	0.991	14.9/21.7
38	Rosmarinic acid	-	26.6	359.0	197.0	0.999	16.2/21.2
39	Ellagic acid	-	27.6	301.0	284.0	0.999	56.9/71.0
40	Cosmosiin	0.043 ± 0.0003	28.2	431.0	269.0	0.998	6.3/9.2
41	Quercitrin	0.314 ± 0.008	29.8	447.0	301.0	0.999	4.8/6.4
42	Astragalin	0.074 ± 0.0008	30.4	447.0	255.0	0.999	6.6/8.2
43	Nicotiflorin	0.082 ± 0.0008	30.6	592.9	255.0/284.0	0.999	11.9/16.7
44	Fisetin	-	30.6	285.0	163.0	0.999	10.1/12.7
45	Daidzein	-	34.0	253.0	223.0	0.999	9.8/11.6
46	Quercetin-D3-IS	N.A.	35.6	304.0	275.9	N.A.	N.A.
47	Quercetin	0.653 ± 0.011	35.7	301.0	272.9	0.999	15.5/19.0
48	Naringenin	6.56 ± 0.257	35.9	270.9	119.0	0.999	2.6/3.9
49	Hesperetin	0.462 ± 0.014	36.7	301.0	136.0/286.0	0.999	7.1/9.1
50	Luteolin	0.297 ± 0.009	36.7	284.8	151.0/175.0	0.999	2.6/4.1
51	Genistein	-	36.9	269.0	135.0	0.999	3.7/5.3
52	Kaempferol	1.286 ± 0,027	37.9	285.0	239.0	0.999	10.2/15.4
53	Apigenin	0.939 ± 0,016	38.2	268.8	151.0/149.0	0.998	1.3/2.0
54	Amentoflavone	0.003 ± 0.0001	39.7	537.0	417.0	0.992	2.8/5.1
55	Chrysin	3.613 ± 0.116	40.5	252.8	145.0/119.0	0.999	1.5/2.8
56	**Acacetin**	**23.604** ± 0.856	40.7	283.0	239.0	0.997	1.5/2.5

-: Not detected, N.A.: Not applicable, R.T.: Retention time, IS: Internal standard, D3: Deuterium isotope 3, M.I. (*m/z):* Molecular ions of the standard analytes (*m*/*z* ratio), FI (*m/z):* Fragment ions, r^2^: Coefficient of determination, LOD/LOQ (µg/L): Limit of detection/quantification. Bold text indicates the highest concentration and the component with this value.

**Table 3:** Structure of the high concentration of phytochemicals found in propolis.

Major Phytochemicals in Propolis	
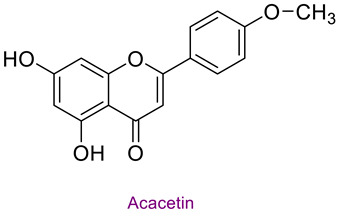	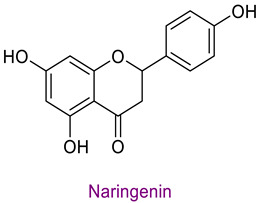
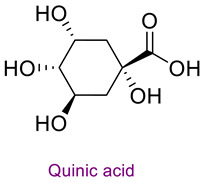	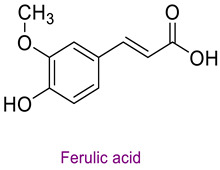
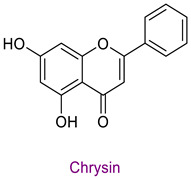	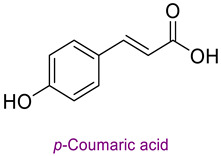
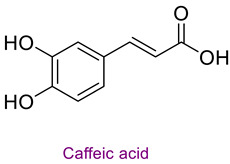	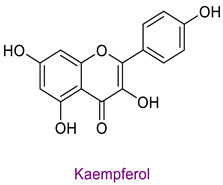

**Table 4:** Mineral content results of propolis.

**Sample**	**Na** (mg/kg)	**Mg** (mg/kg)	**Al** (mg/kg)	**K** (mg/kg)	**Ca** (mg/kg)	**V** (mg/kg)
**Propolis**	124.3 ± 4.2	710.8 ± 13.7	1066.2 ± 27.3	1521.4 ± 33.1	483.7 ± 11.9	1.8 ± 0.03
**Sample**	**Cr** (mg/kg)	**Mn** (mg/kg)	**Fe** (mg/kg)	**Co** (mg/kg)	**Ni** (mg/kg)	**Cu** (mg/kg)
**Propolis**	2.3 ± 0.05	31.7 ± 2.19	1068.2 ± 33.5	0.6 ± 0.002	2.8 ± 0.02	2.2 ± 0.03
**Sample**	**Zn** (mg/kg)	**As** (µg/kg)	**Ag** (µg/kg)	**Cd** (µg/kg)	**Pb** (µg/kg)	**Se** (µg/kg)
**Propolis**	73.4 ± 2.23	<LOD	<LOD	1.1 ± 3.15	18.6 ± 0.02	<LOD

The three parallel measurements’ mean ± standard deviation is the result. LOD: Limit of detection.

**Table 5:** Total phenolic and flavonoid content results of the propolis sample.

Sample	Total Phenolics (mg GAE/g)	Total Flavonoids (mg QE/g)
**Propolis**	215.14 ± 1.19	79.11 ± 0.39

**Table 6:** Results of antioxidant-reducing assays of propolis (µg/mL).

**Antioxidants and Sample**	**Fe^3+^ Reducing**		**Cu^2+^ Reducing**		**Fe^3+^-TPTZ Reducing**	
	**λ _700_**	**r^2^**	**λ_450_**	**r^2^**	**λ _593_**	**r^2^**
**BHA**	2.123 ± 0.020	0.9914	1.400 ± 0.051	0.9472	0.942 ± 0.007	0.9830
**BHT**	1.464 ± 0.014	0.9797	1.342 ± 0.007	0.9775	0.799 ± 0.007	0.9960
**Trolox**	1.270 ± 0.014	0.9905	0.706 ± 0.017	0.9759	0.883 ± 0.020	0.9895
**α-Tocopherol**	1.912 ± 0.084	0.9871	1.133 ± 0.086	0.9885	1.113 ± 0.010	0.9978
**Propolis**	0.940 ± 0.029	0.9907	1.183 ± 0.041	0.9858	0.963 ± 0.024	0.9759

All values are averages of three parallel observations (*n* = 3) and are shown as mean SD (*p* < 0.05 is considered significant).

**Table 7:** DPPH^•^ and ABTS^•+^ scavenging assays results of propolis (µg/mL).

Antioxidants and Sample	DPPH^•^ Scavenging		ABTS^•+^ Scavenging	
	IC_50_	r^2^	IC_50_	r^2^
**BHA**	9.900 ± 0.03	0.9618	4.521 ± 0.03	0.9930
**BHT**	14.140 ± 0.04	0.9935	5.812 ± 0.03	0.9805
**Trolox**	6.026 ± 0.03	0.9429	4.813 ± 0.02	0.9749
**α-Tocopherol**	9.240 ± 0.03	0.9748	7.304 ± 0.03	0.9982
**Propolis**	16.700 ± 0.09	0.9851	8.010 ± 0.05	0.9806

All values are averages of three parallel observations (*n* = 3) and are shown as mean SD (*p* < 0.05 is considered significant).

**Table 8:** Inhibition values of propolis sample against hCA I and hCA II isoenzymes (IC_50_: µg/mL).

Sample and Standard	hCA I		hCA II	
	IC_50_	r^2^	IC_50_	r^2^
**Propolis**	13.2	0.9799	14.5	0.9889
Acetazolamide *	7.19	0.9913	8.15	0.9983

* Acetazolamide was employed as a standard inhibitor of hCA I and hCA II.

**Table 9:** Inhibition values of propolis sample against α-Glycosidase, AChE and BChE isoenzymes (IC_50_: µg/mL).

Sample and Standard	α-Glycosidase		AChE		BChE	
	IC_50_	r^2^	IC_50_	r^2^	IC_50_	r^2^
**Propolis**	5.72	0.9799	5.17	0.9889	7.50	0.9895
Acarbose *	15.95	0.9892	-	-	-	-
Tacrine **	-	-	8.15	0.9983	8.15	0.9983

* Acarbose was used as a standard inhibitor for the α-glycosidase enzyme. ** Tacrine was used as a standard inhibitor for AChE and BChE enzymes.

## Data Availability

All data are available in the study. For questions, the corresponding author can be contacted.
